# Metastatic Malignant Melanoma Mimicking Mammary Mass: A Rare Presentation

**DOI:** 10.7759/cureus.8354

**Published:** 2020-05-29

**Authors:** Ankit Jain, Chellappa Vijayakumar, Uday Kumbhar, Sudharsanan Sundaramurthi, Gopal Balasubramanian

**Affiliations:** 1 Surgery, Jawaharlal Institute of Postgraduate Medical Education and Research (JIPMER), Puducherry, IND

**Keywords:** melanoma, metastsis, carcinoma breast

## Abstract

Breast lump in perimenopausal women is considered a primary malignancy unless proved otherwise. Metastasis to the breast from extramammary sites is rare. Malignant melanoma (MM) is known for its ability to spread to distant sites, which can be both hematogenous and lymphatic. The common sites are skin, lung, liver, brain, etc. However, reports of melanoma metastasizing to the breast are rare.

We present a case of 50-year-old female patient, who underwent wide local excision and split skin grafting for MM right leg. She did not undergo any adjuvant therapy and one year later presented to us with a solitary lump occupying the upper inner quadrant of the right breast. The breast lump turned out to be metastatic deposit from MM based on the presence of melanin in cells on fine needle aspiration cytology (FNAC). Therefore, breast lump in perimenopausal is not always a primary malignancy, and differential diagnosis should also include metastatic tumors.

## Introduction

The breast is a site for numerous primary benign and malignant lesions. Metastasis to the breast is rare, commonly from opposite mammary tissue, and rarely from the extramammary sites. It is seen in approximately 1.3%-2.7% of all the malignant breast tumors [1]. Malignant melanoma (MM) is known for its ability to spread to distant sites, seen in 20% of cases, which can be both hematogenous and lymphatic [1]. The most common primary malignancies metastasizing to the breast are lymphomas, melanomas, rhabdomyosarcomas, lung and ovarian tumors [1-3]. We report one such rare case of MM of a leg with metastasis to breast. 

## Case presentation

**Figure 1:**
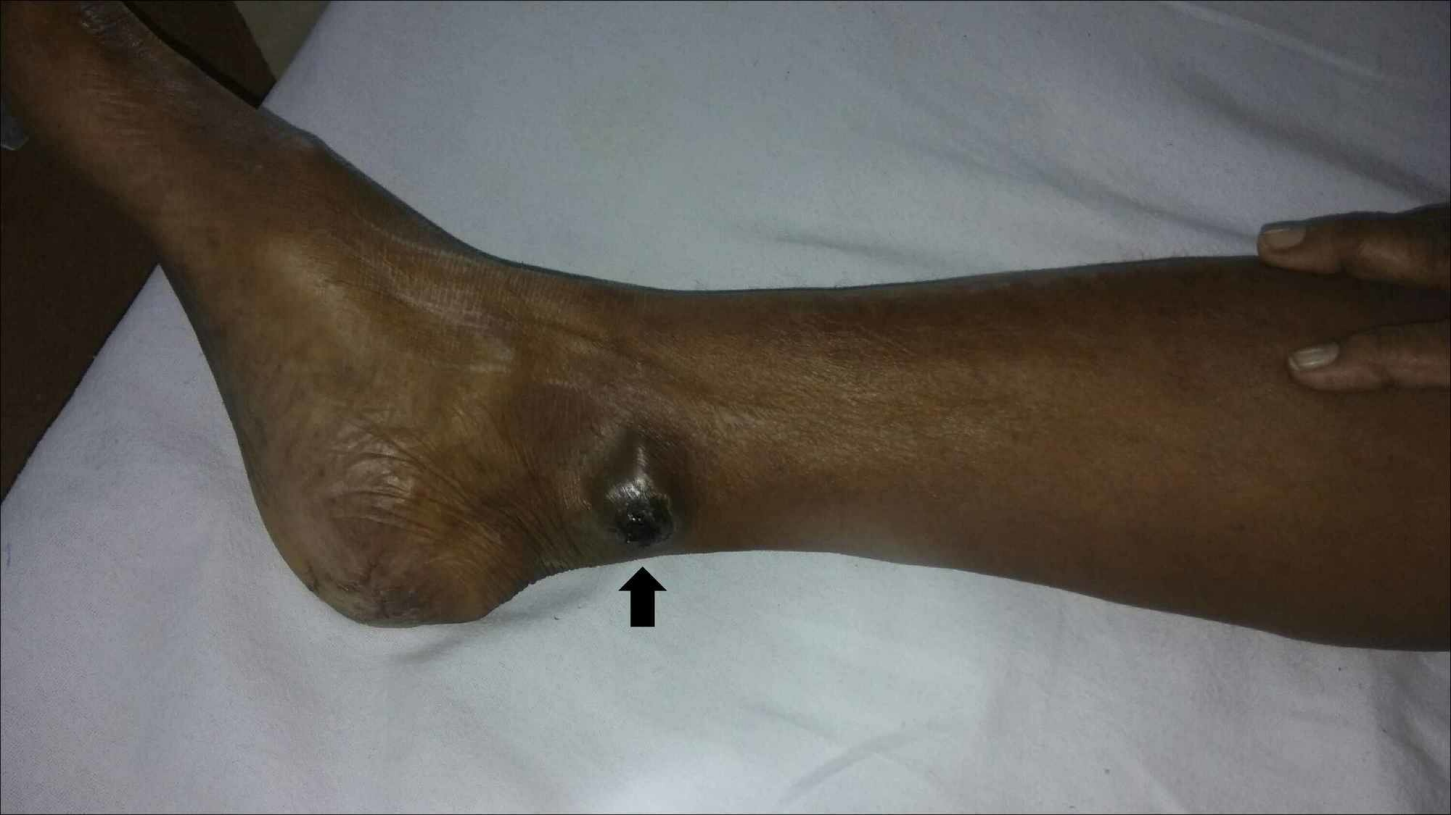
Clinical picture of malignant melanoma in the right leg (primary site - black arrow).

A 50-year-old female patient with a known case of MM in the right leg was treated with wide local excision and split skin graft two years back (Figure 1).

**Figure 2:**
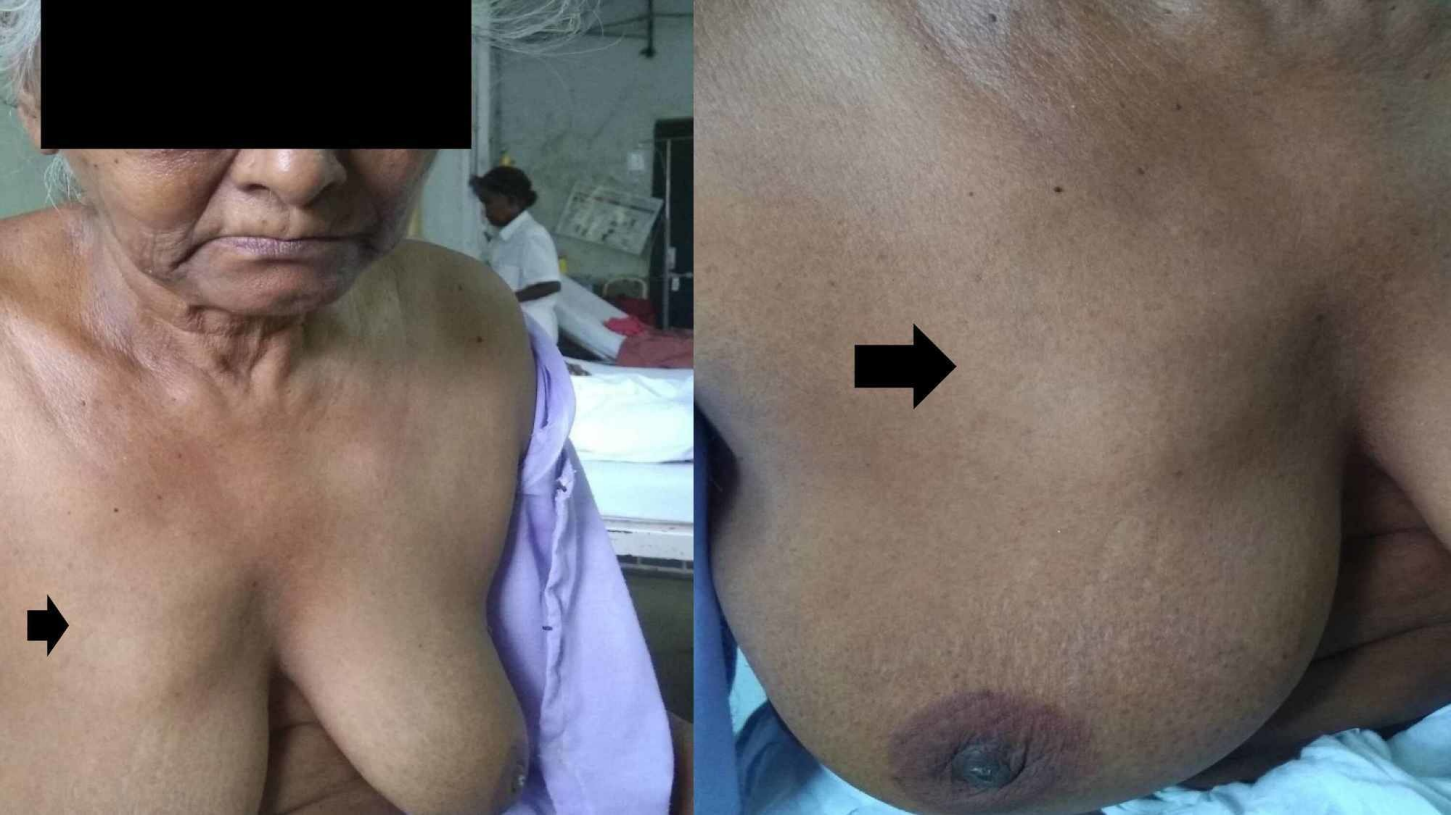
Clinical picture of solitary right breast mass (upper inner quadrant - black arrow).

The patient was lost to follow-up and did not undergo any further treatment. One year back, she presented with a complaint of a lump in the right breast for one month. There was no history of lump or skin lesion in the right leg or anywhere else in the body including left breast. There were no neurological complaints. On examination, a single 5x6 cm, firm, nodular mass with well-defined margins, not fixed to underlying muscles, was palpable in the upper inner quadrant of the right breast. There were no overlying skin changes, nipple discharge or nipple retraction (Figure 2). 

**Figure 3:**
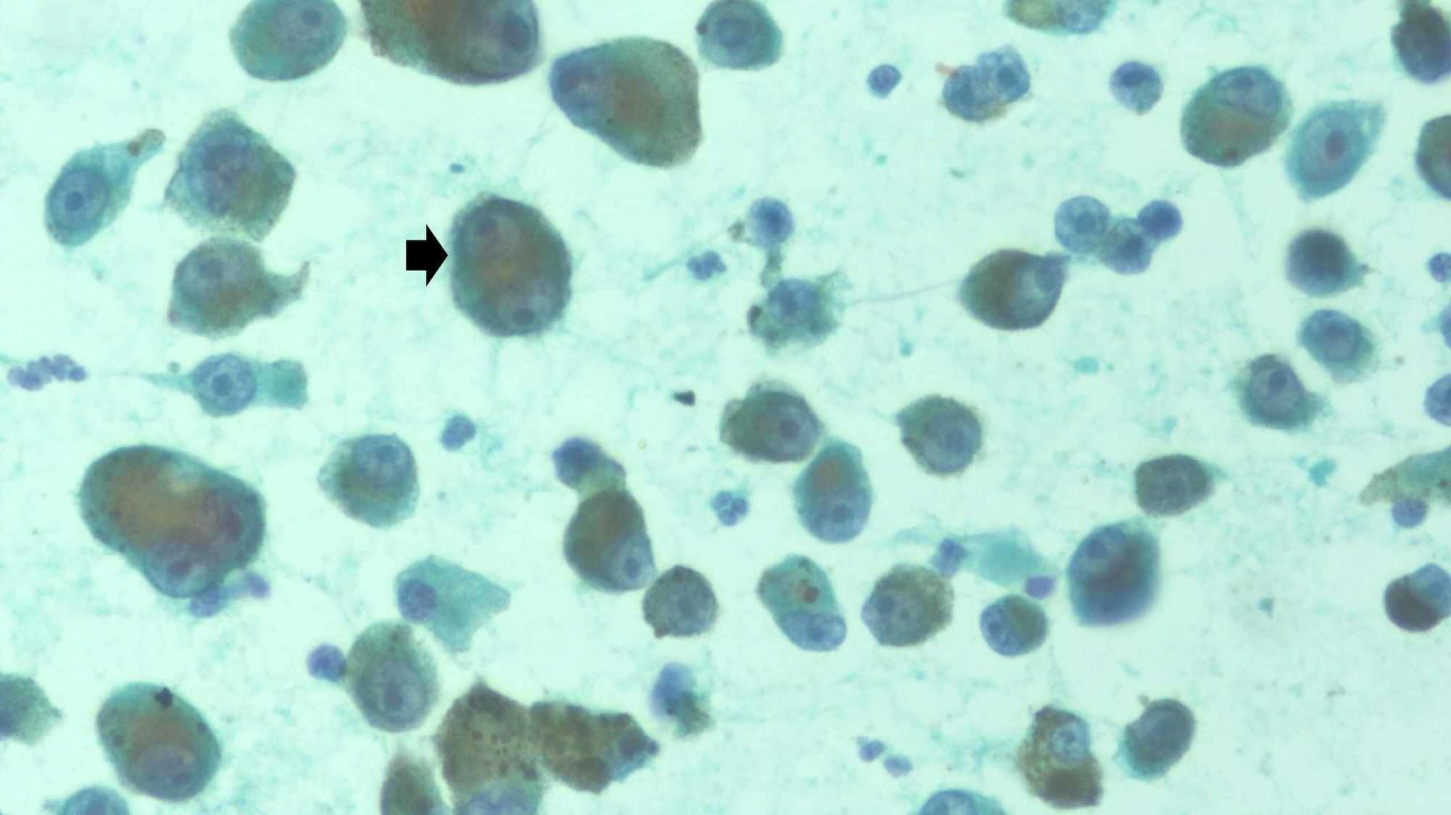
Microscopic examination of FNAC showing most of the tumor cells containing melanin pigments in the cytoplasm, confirming MM (black arrow). The background showed small scattered lymphoid cells (X400). FNAC: fine needle aspiration cytology; MM: malignant melanoma

There was no palpable lump in the opposite breast, both axillae and the neck region. There was no skin nodule palpable in the right leg or inguinal lymphadenopathy. Mammography was suggestive of an oval opacity with well-defined edges without calcifications or architectural distortion. Fine needle aspiration cytology (FNAC) was performed on the lump, which showed dyscohesive scattered pleomorphic tumor cells with abundant cytoplasm and eccentrically placed nucleoli. Most of the tumor cells showed melanin pigment in the cytoplasm, thus confirming metastasis from MM (Figure 3).

The case was discussed with the multidisciplinary team (MDT) for further management. MDT advised the positron emission tomography (PET) scan for metastatic workup. But again, the patient did not follow up and expired after one year with disseminated bony metastases.

## Discussion

MM constitutes less than 2% of skin cancers, even though it is the most common cause of skin cancer-related mortality in the world. It is notorious for its ability to spread to distant sites through hematogenous or lymphatic routes, seen in 20% cases [2]. Most common sites are skin, lung, liver, brain, etc. [1,2]. However, reports of melanoma metastasizing to the breast are rarely found in the literature. Breast involvement by MM can have varied presentations, such as (i) primary MM of the breast skin; (ii) primary MM of the breast glandular tissue; (iii) in-transit metastases to breast tissue and skin and (iv) metastasis to the breast parenchyma from distant primary [4].

Primary carcinoma is the most common malignancy of the breast. A lady, presenting with a breast lump, needs to be evaluated and worked up for ruling out primary breast malignancy. In such a scenario, differentiating primary lesions from metastatic breast lesions is very important. The triple assessment for breast cancer can be helpful in this. A detailed history can be taken for any pre-existing malignancy or a current lump or symptoms not related to breast. Patients with metastasis to the breast are younger and generally premenopausal [2]. In more than 50% of cases, metastasis is seen in the outer quadrant because of good vascularity and the presence of more glandular tissue [5]. The outer half of the breast is also the most common site of the primary malignancy. Therefore, the site cannot differentiate between primary and metastatic tumors. Just like primary malignancy, a unilateral solitary lesion is the more common presentation [6]. In our case, the lump was unilateral and solitary, but was located in the upper inner quadrant.

On sonography, metastatic lesions can be round to oval with circumscribed or irregular margins, with or without posterior acoustic shadowing [2,6]. On mammography, the most common finding is well-defined nodular opacities without microcalcification, architectural distortion or skin involvement [6]. A definitive diagnosis is reached with pathological examination such as FNAC or biopsy. On histological examination, MM has varied microscopic morphological features that can confuse with other malignant tumors [7]. The presence of melanin pigment within tumor cells is a definitive but very rare feature [2]. Thus, immunohistochemistry (IHC) helps in reaching the correct diagnosis due to the presence of S100 protein, Melan-A and HMB-45 along with no expression for cytokeratin staining. This helps in differentiating MM from other malignant tumors. Therefore, a combination of thorough clinical history and tissue diagnosis is required to differentiate metastasis from primary breast lesions. In our case, mammographic features and the presence of melanin in cells on FNAC were diagnostic of secondaries from MM; therefore, IHC study was not done.

MM with metastasis has a poor prognosis with overall survival ranging from 4.7 to 11 months [8]. Treatment options for MM include close observation, surgical resection of isolated metastases, chemotherapy, radiation therapy and immunotherapy. In the past, dacarbazine and interleukin 2 (IL-2) therapy were used either alone or in combination with other drugs with not much improvement in overall survival [2]. However, the use of targeted therapy and immunotherapy shows survival benefits in patients. Vemurafenib, a BRAF inhibitor, and ipilimumab, an anti-cytotoxic T-lymphocyte antigen 4 antibody, are the first agents that demonstrated a survival benefit [4]. Radiation therapy plays a limited role as radio-sensitivity of melanoma cells is extremely low. Patients with limited metastasis can undergo metastasectomy, which offers both palliation and survival benefit. However, patients rarely present with limited metastasis. Unfortunately, this patient expired within one year of diagnosis of breast metastasis without any treatment, due to loss from follow-up.

## Conclusions

Although primary malignancy is the most common tumor of breast tissue, the possibility of metastasis from extramammary malignancy like MM should be kept in mind while evaluating a patient with a breast lump. Due to non-discriminatory clinical and radiological findings, a crucial role is played by detailed history and histopathological examination in its diagnosis.
